# Indocyanine green fluorescence imaging to assess bowel perfusion during totally laparoscopic surgery for colon cancer

**DOI:** 10.1186/s12893-020-00745-4

**Published:** 2020-05-13

**Authors:** Hao Su, Hongliang Wu, Mandula Bao, Shou Luo, Xuewei Wang, Chuanduo Zhao, Qian Liu, Xishan Wang, Zhixiang Zhou, Haitao Zhou

**Affiliations:** 1 Department of Colorectal Surgery, National Cancer Center/ National Clinical Research Center for Cancer/ Cancer Hospital, Chinese Academy of Medical Science and Peking Union Medical College, No. 17, Pan Jia Yuan Nan Li, Chaoyang District Beijing, 100021 People’s Republic of China; 2 Department of Anesthesiology, National Cancer Center/ National Clinical Research Center for Cancer/ Cancer Hospital, Chinese Academy of Medical Science and Peking Union Medical College, Beijing, 100021 China

**Keywords:** Indocyanine green, Fluorescence imaging, Totally laparoscopy, Bowel perfusion, Anastomotic leak

## Abstract

**Background:**

To retrospectively evaluate the feasibility and safety of intraoperative assessment of bowel perfusion in totally laparoscopic surgery for colon cancer using indocyanine green fluorescence imaging (IGFI).

**Methods:**

From October 2017 to June 2019, consecutive patients with colon cancer who underwent totally laparoscopic surgery were enrolled retrospectively and grouped into the IGFI group (*n* = 84) and control group (*n* = 105). In the IGFI group, indocyanine green (ICG) was injected intravenously, and the bowel perfusion was observed using a fluorescence camera system prior to and after completion of the anastomosis.

**Results:**

The two groups were demographically comparable. The IGFI group exhibited a significantly shorter operative time (*p* = 0.0374) while intraoperative blood loss did not significantly differ among the groups (*p* = 0.062). In the IGFI group, average time to perfusion fluorescence was 48.4 ± 14.0 s after ICG injection, and four patients (4.8%) were required to choose a more proximal point of resection due to the lack of adequate fluorescence at the point previously selected. There were no differences in terms of pathological outcomes, postoperative recovery and the postoperative complication rates between the groups (*p*>0.05).

**Conclusion:**

IGFI shows promise as a safe and feasible tool to assess bowel perfusion during a totally laparoscopic surgery for colon cancer and may reduce the operative time.

## Background

Colorectal cancer (CRC) has rapidly increased and has become the third most commonly diagnosed type of cancer and second leading cause of death worldwide [1]. Surgery remains the standard treatment for colon cancer with curative intent, and recently, totally laparoscopic surgery for colon cancer with intracorporeal anastomosis has been expected to be less invasive, with earlier postoperative recovery and lower complication rates [2, 3].

Anastomotic leak is still one of the most dreaded postoperative complications in colonic surgery, ranging from 1 to 20% [4]. Among factors identified as possible causes of anastomotic leakage, inadequate anastomotic vascular perfusion seems to have a significant impact on the healing of an anastomosis [5, 6]. However, the evaluation of anastomotic vascular perfusion in totally laparoscopic surgery seem to be more difficult. It usually depends on the surgeon’s visual judgment of the color change or pulsation of the small blood vessels in the colon wall, which is supposed to underestimate the risk of anastomotic leakage.

Indocyanine green fluorescence imaging (IGFI) is a real-time method to evaluate the organ perfusion, based on direct visualization of the fluorescence emitted by indocyanine green (ICG) under near-infrared (NIR) light after the intravenous injection [7]. This technique has been widely used in various branches of surgical medicine including plastic, cardiothoracic, hepatobiliary, and gastrointestinal surgeries [8, 9].

Therefore, this study aimed to present our results of IGFI on evaluating bowel perfusion during a totally laparoscopic surgery for colon cancer and compare the outcomes with conventional totally laparoscopic surgery.

## Methods

### Patients

From October 2017 to June 2019, consecutive patients diagnosed with colon cancer who underwent totally laparoscopic surgery by a single surgeon in our hospital were enrolled retrospectively. Eligible patients were 18–80 years of age, body mass index (BMI) between 18.5 and 30 kg/m^2^ and with a pathological diagnosis of colon adenocarcinoma by colonoscopy. Patients with history of past colonic surgery, multiple colorectal primary carcinomas, distant metastasis, and allergic hypersensitivity to ICG were excluded. Because this study aimed to evaluate the effect of IGFI in totally laparoscopic colonic resection, tumors located < 30 cm from the anal margin by enteroscopy were also excluded. They were divided into 2 groups: the IGFI group (84 patients), who underwent totally laparoscopic surgery using IGFI, and the control group (105 patients), who underwent conventional totally laparoscopic surgery. The study was conducted in accordance with the principles of the Declaration of Helsinki. The procedure used during this study was explained to all patients in detail prior to surgery, and every patient provided written informed consent for surgery.

Patient demographics were collected including age, gender, body mass index (BMI), American Society of Anesthesiologists (ASA) score, tumor location, previous abdominal operation history, preoperative chemotherapy. Collected surgical factors included operative time, estimated blood loss, removal method of the specimen, and pathological outcomes. Postoperative complications including anastomotic leak, anastomotic stenosis, bleeding, urinary tract infection, urinary retention, bowel obstruction, incisional infection, and ICG allergy were collected. Anastomotic leak was defined as a defect at the anastomotic site leading to a communication between intra- and extra-luminal compartments proven clinically and radiologically occurring within 60 days postoperatively. Reoperation and readmission rates were also measured.

### Surgical procedures

The IGFI group used the system provided by opto-cam 2100 (Optomedic, Guangdong, China). This device can be used for standard laparoscopic visible imaging mode and can be switched to NIR fluorescence mode by means of button control on the camera head, on the stack console, or via the foot pedal. ICG (25 mg, Eisai, Tokyo, JP) was diluted in 10 ml of distilled water, and a minimum dose of 3 ml was rapidly injected into the peripheral blood vessels one at a time just before fluorescence observation. The control group used the conventional HD laparoscopic procedure system.

Under general anesthesia, all patients were placed in the supine lithotomy position, and a five- or four-port technique was used. Relevant colons were mobilized from their retroperitoneal attachments according to the principle of complete mesocolic excision (CME). Vessels were isolated and ligated with a laparoscopic blunt tip vessel sealer or divider. D3 LN dissection was performed in all patients.

**Fig. 1:**
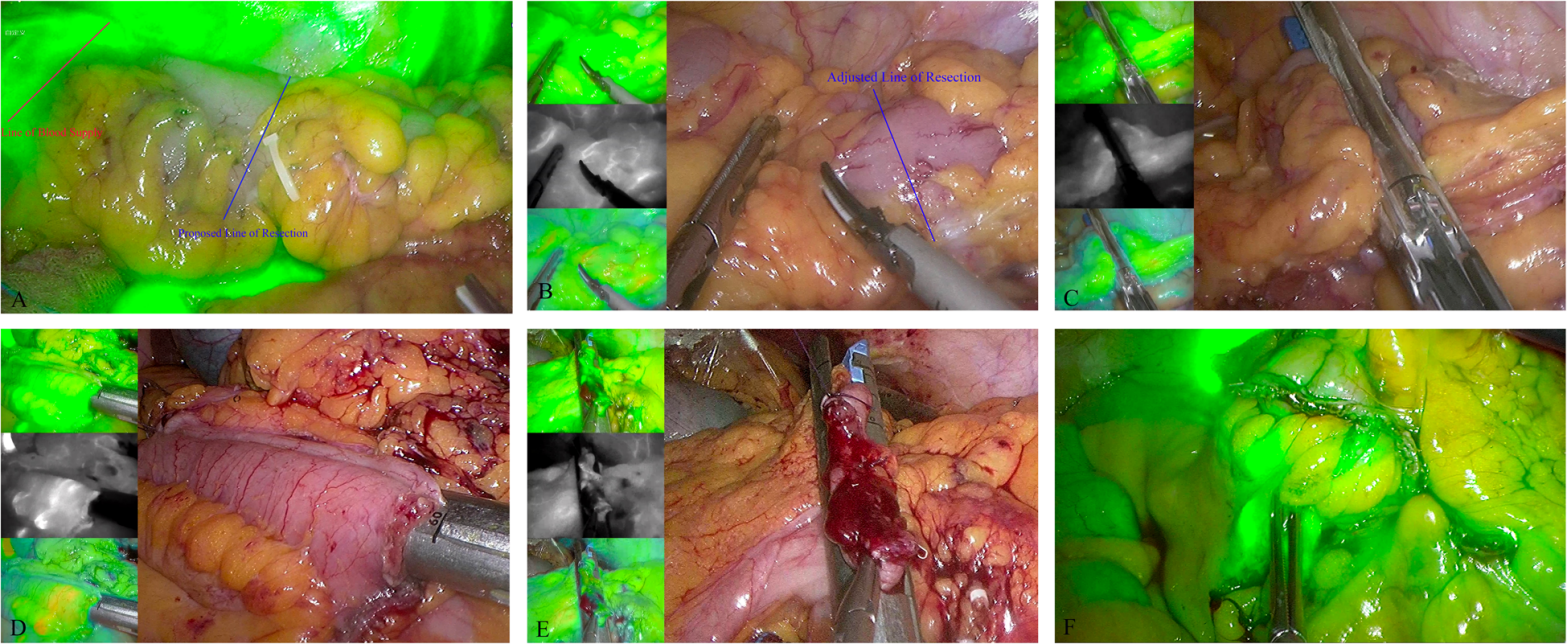
The surgical procedures of overlapped delta-shaped anastomosis using IGFI. **a** the perfusion of the bowel was assessed; **b** further “re-resection” up to a “fluorescent” portion; **c** the colon is transected within an area of well- perfused tissue; **d** the two broken ends of the intestines are joined; **e** the enterocolotomy is closed; and **f** the perfusion of anastomosis was assessed

All patients underwent overlapped delta-shaped anastomosis: In the IGFI group, a dose of 3 ml ICG was intravenously injected through a peripheral vein after dividing the mesentery at approximately 10 cm from the tumor. Bowel perfusion was subjectively assessed and recorded by the surgical team in real time (Fig. 1a), and patients would receive further “re-resection” up to a “fluorescent” portion if the perfusion of the bowel was insufficient (Fig. 1b). The colon was then divided within an area of well-perfused tissue with 60 mm linear staplers (Fig. 1c). Resected specimens were collected and stored in specimen bags immediately. The proximal and distal intestines were approximated and joined for an overlapped side-to-side anastomosis using a 60 mm linear stapler (Fig. 1d). Three interrupted sutures were placed to pull the enterotomy, which was then closed by applying another 60 mm linear stapler (Fig. 1e). The digestive tract reconstruction was completed, and after completing the anastomosis, another dose of 3 ml ICG was injected and second evaluation of perfusion was made (Fig. 1f). The control group used the same anastomotic procedure without IGFI and the vascular anastomotic perfusion was assessed by surgeon’s naked eyes.

For all patients, the specimen was removed either from the natural orifice (anus or vagina), abdominal scar of the previous surgery, or 5–6 cm from the Pfannenstiel incision made above the symphysis pubis at the border of the pubic hair.

### Statistical analysis

Statistical analysis was performed with SPSS software version 20.0 for Windows (SPSS Inc., Chicago, IL, USA). Quantitative variables are presented as mean and standard deviations and qualitative data as number and its percentage. Quantitative variables are compared with the Student t-test and qualitative variables are compared with the χ^2^-test. *P*-values of less than 0.05 were considered statistically significant.

## Results

### General data

Patient demographics are presented in Table 1. There were no significant differences in terms of age, gender, BMI, ASA scores, tumor location, previous abdominal operation history and preoperative chemotherapy between the IGFI and control groups (*p*>0.05). The IFGI group had a tendency towards obesity while without statistical significance (*p* = 0.066). There were more patients with right colon cancer in the control group although not statistically significant (*p* = 0.761). The percentage of patients per ASA scores (1,2,3,4) were comparable between the two groups (*p* = 0.279).

**Table 1:** Clinical characteristics of patients

	IGFI group (*n* = 84)	Control group (*n* = 105)	*P* value
Gender, *n* (%)			0.514
Male	48 (57.1)	55 (52.4)	
Female	36 (42.9)	50 (47.6)	
Age, yr, mean ± SD	59.1 ± 11.1	60.2 ± 9.8	0.478
BMI, kg/m^2^, mean ± SD	24.6 ± 3.4	23.8 ± 2.7	0.066
ASA score, *n* (%)			0.279
1	28 (33.3)	45 (42.9)	
2	50 (59.5)	50 (47.6)	
3	6 (7.1)	10 (9.5)	
4	0	0	
Previous abdominal operation, *n* (%)			0.083
Yes	17 (20.0)	33 (31.4)	
No	67 (80.0)	72 (68.6)	
Preoperative neoadjuvant therapy, *n* (%)			
NACT	14 (16.7)	30 (28.6)	0.054
No	70 (83.3)	75 (71.4)	
Tumor location, *n* (%)			0.761
Ileocecal junction	20 (23.8)	25 (23.8)	
Ascending colon	25 (29.8)	28 (26.7)	
Hepatic flexure	9 (10.7)	18 (17.1)	
Transverse colon	12 (14.3)	10 (9.5)	
Splenic flexure	5 (5.9)	6 (5.7)	
Descending colon	8 (9.5)	8 (7.6)	
High sigmoid colon	5 (5.9)	10 (9.5)	

### Surgical and pathological findings

All patients in this study underwent totally laparoscopic surgery successfully, and surgical and pathological data are presented in Table 2. The mean operation time for the IFGI group was 125.8 ± 34.9 min, significantly shorter than the 136.6 ± 35.9 min for the control group (*p* = 0.037). The intraoperative blood loss (45.7 ± 41.7 ml vs. 58.7 ± 53.7 ml, *p* = 0.062) were similar between groups. In the IFGI group, the mean time to perfusion fluorescence was 48.4 ± 14.0 s after ICG injection. Four patients (4.8%) in the IFGI group who had left colectomies underwent further “re-resection” up to the “fluorescent” portion due to insufficient bowel perfusion, and all of these plan changes occurred while transecting the proximal margin. Majority of the specimen was removed from the Pfannenstiel incision, and no intraoperative or anesthetic complications occurred. All patients were pathologically diagnosed with negative resection margin, and pathological outcomes were comparable between the two groups.

**Table 2:** Surgical and pathological outcomes of patients

	IGFI group (*n* = 84)	Control group (*n* = 105)	*P* value
Operation time, min, mean ± SD	125.8 ± 34.9	136.6 ± 35.9	0.037
Intraoperative blood loss, ml, mean ± SD	45.7 ± 41.7	58.7 ± 53.7	0.062
Surgical Procedure, *n* (%)			0.747
Right colectomy	55 (65.5)	72 (68.6)	
Transverse colectomy	10 (11.9)	9 (8.6)	
Left colectomy	19 (22.6)	24 (22.9)	
Removal method of the specimen, *n* (%)			0.285
Natural orifice	5 (6.0)	8 (7.6)	
Abdominal scar	15 (17.9)	28 (26.7)	
Pfannenstiel incision	64 (76.2)	69 (65.7)	
The length of tumor, cm, mean ± SD	3.8 ± 1.5	3.6 ± 1.8	0.446
Proximal resection margin, cm, mean ± SD	11.1 ± 3.7	10.2 ± 2.9	0.088
Distal resection margin, cm, mean ± SD	9.7 ± 3.2	9.2 ± 1.9	0.190
Number of lymph nodes retrieved, mean ± SD	23.1 ± 9.4	21.8 ± 8.5	0.307
pTNM stage, *n* (%)			0.555
I	9 (10.7)	15 (14.3)	
II	32 (38.1)	44 (41.9)	
III	43 (51.2)	46 (43.8)	

### Postoperative recovery and complications

In this study, no significant differences were observed between the groups in terms of the postoperative hospitalization and cost, respectively. With a follow-up until December, 2019, the overall rate of complications was comparable between the groups (11.9% vs. 12.4%, *p* = 0.921). The most common complication was incision infection in both groups, and no patient experienced anastomotic leak. One patient in the IFGI group suffered abdominal pain on postoperative day 5, and the computed tomography (CT) scan found a pelvic fluid collection, which was drained revealing serosanguinous fluid and thus were not defined as anastomotic leak. No side effects or allergic reactions related to ICG injection were observed. Both groups had one patient who suffered a postoperative anastomotic bleeding that was successfully treated conservatively (Table 3). No patient was lost to follow-up, and none of them experienced recurrence or death during the follow-up period. All complications were resolved successfully.

**Table 3:** Postoperative recovery and complications

	IFGI group (*n* = 84)	Control group (*n* = 105)	*P* value
Postoperative hospitalization, days, mean ± SD	5.7 ± 1.4	6.0 ± 1.5	0.139
Hospitalization cost, USD, mean ± SD	9661.9 ± 987.7	9520.0 ± 854.4	0.291
Postoperative complications, *n* (%)	10 (11.9)	13 (12.4)	0.921
Anastomotic leak	0	0	
Anastomotic stenosis	0	0	
Anastomotic bleeding	1 (1.2)	1 (1.0)	
Urinary tract infection	1 (1.2)	2 (1.9)	
Urinary retention	1 (1.2)	1 (1.0)	
Bowel obstruction	1 (1.2)	1 (1.0)	
Incisional infection	6 (7.1)	8 (7.6)	
ICG allergy	0	0	
Reoperation, *n* (%)	0	0	–
Readmission, *n* (%)	0	0	–
Mortality, *n* (%)	0	0	–

## Discussion

Totally laparoscopic colectomy is accepted and performed as a method, showing improvement in the surgical treatment for colon cancer. Many obvious advantages of totally laparoscopic colectomy have been revealed as compared with conventional laparoscopic-assisted colectomy. Carmelo Magistro et al. thought direct manipulation of the bowel trait harboring the lesion is minimized and the entirely intracorporeal procedure decreased the traction of the mesentery and the risk of anastomotic twist [10]. Francesco Roscio et al. thought that totally laparoscopic surgery would be an ideal treatment for patients with higher BMI, because it prevented extensive incisions for the extraction of large specimens through very thick abdominal walls and reduced the risk of microlacerations during the exteriorization of heavy and short mesenteries [11]. Ilknur Erguner et al. thought that totally laparoscopic surgery avoided ischemia–reperfusion of the colon during extracorporeal anastomosis for a minimum of 5–10 min and provided a free specimen extraction site such as suprapubic incision or the natural orifices, which would offer less adhesions, less incisional hernia, and better cosmesis [12]. Our previous studies also found that totally laparoscopic surgery for colon cancer had the advantages of less postoperative pain and earlier time to first flatus, which promises a safe and feasible procedure with satisfactory short-term outcomes [13, 14].

However, different from conventional extracorporeal anastomosis, the evaluation of bowel perfusion under laparoscopy may be difficult for beginners due to the lack of stereoscopic vision, while the judgment of perfusion may be a key factor for the healing of an anastomosis in colonic surgeries. Generally, surgeons assessed the vascular anastomotic perfusion by active bleeding from the resection margin, palpable pulsation in the mesentery, or lack of discoloration, which was subjective, highly unreliable, and time-consuming [15]. Doppler ultrasound, laser Doppler flowmetry, angiography, and oxygen spectroscopy are thought to be reliable methods to evaluate bowel perfusion, which were not widely used in the surgical field due to the price of equipment, technical difficulties, and lack of reproducibility [16, 17].

ICG is a sterile, anionic, water-soluble solution but with relatively hydrophobic and tricarbocyanine molecules with the weight of 775 Da, which absorbs light between 790 and 805 nm and re-emits it with an excitation wavelength of 835 nm presented as a fluorophore in response to NIR irradiation [17]. After an intravenous injection, ICG rapidly and extensively binds to the plasma protein, with minimal leakage into the interstitium. With the half-life of 3–5 min, ICG is cleared by the liver in 15–20 min into bile with no known metabolites. Intravenous use is reported to be very safe generally, and cases of vasovagal or allergic reactions such as anaphylactic shock, hypotension, tachycardia, dyspnea, or urticaria are extremely rare. These properties make ICG an ideal agent for the acquisition of high-quality images of both the circulatory and lymphatic systems [7–9].

The recently developed IGFI facilitates easy performance of intraoperative fluorescence angiography and has been used to evaluate the real-time perfusion of the resection margin during a laparoscopic surgery [18–20]. It was reported in some meta-analysis that ICG fluorescence imaging was an effective tool to assess anastomotic perfusion and reduced anastomotic leakage rates in patients undergoing colorectal resection [21, 22]. Some multicenter randomized controlled trials also found that intraoperative ICG fluorescent angiography could effectively assess vascularization of the anastomosis and lead to a reduction in anastomotic leak in in colorectal surgery [23, 24].

Based on these studies, we applied the technique of IGFI in totally laparoscopic surgery for colon cancer and evaluate the feasibility, safety, and short-term outcomes. Some studies assessed the perfusion of colonic tissue based on the integrity of the mucosal aspect of the completed anastomosis using fluorescence angiography via proctoscopy [25]. The conversion of different devices added the total operation time. During a totally laparoscopic surgery for colon cancer, we used IGFI to evaluate the bowel perfusion only under laparoscopy, and the mean operation time is 125.8 ± 34.9 min, which is significantly shorter than the control group, and we attributed this difference to the fast and accurate judgment of bowel perfusion and the simplicity of this procedure. Moreover, the mean time to perfusion fluorescence in this study was 48.4 ± 14.0 s after the injection of ICG. Therefore, we thought the overall operation time was not prolonged due to the application of this new technique, which conversely shortened the total time due to the quick operation during the selection of resection margin. In fact, during totally laparoscopic surgery for colon cancer, we found that IGFI was easy to implement with a short learning curve due to the similarity of this device with a standard laparoscope.

Our results indicated that the assessment of perfusion at the proximal and distal resection margins was associated with revision of the surgical plan in nearly 4.8% of patients and the percentage was not low during a totally laparoscopic surgery. We thought that the microperfusion deficiency of the transected bowel and planned site of anastomosis could not be found by conventional methods of assessment and may be not entirely reliable. Further, patients who underwent further “re-resection” up to a “fluorescent” portion due to insufficient bowel perfusion in this study were those with left colectomies. With regard to the reason, previous studies found the blood pressure of marginal artery was reduced by ≥30% after the blockage of the left colic artery and unstable blood flow to the left colon occurred after IMA ligation in approximately 10% of patients, which could increase the rate of insufficient perfusion [26, 27]. Therefore, we confirmed that insufficient perfusion of the bowel may more frequently occur in patients with left colon cancer, and we should focus more on the bowel perfusion in totally laparoscopic left hemicolectomy, especially for the proximal margin.

IGFI was used to guarantee a reliable anastomosis, in order to prevent complications related to the anastomosis. Previous studies have clearly demonstrated that IGFI can reduce anastomotic complications, and the incidence of complications in this study was 11.9%, which was similar to that of other studies [25, 28]. Fortunately, no patient suffered anastomotic leak and only one patient suffered anastomosis-related bleeding that was successfully treated conservatively. Benign anastomosis-related stricture after the colorectal anastomosis occurs in some patients, and its occurrence is not rare. Preoperative radiation, anastomosis-related ischemia, leakage, and the anastomosis technique are all thought to be related to the development of anastomotic stricture [29]. In this study, no patient suffered from anastomotic stricture, which can be attributed to a reliable intraoperative real-time blood flow evaluation by IGFI and advanced overlapped delta-shaped anastomosis, in which the staple line in the anastomotic stoma appeared as a curving obtuse triangle after the digestive tract reconstruction.

## Conclusion

This preliminary study demonstrates that IGFI is a promising intraoperative tool for the assessment of bowel perfusion and can guarantee a rapid and reliable anastomosis during a totally laparoscopic colectomy. Further prospective randomized controlled trials from multiple centers with larger sample sizes and longer follow-up periods may confirm that IGFI can decrease the rate of anastomotic leak and thereby improve outcomes of colorectal cancer surgery.

## Data Availability

The datasets generated and/or analysed during the current study are not publicly available due to the data is confidential patient data but are available from the corresponding author on reasonable request.

## Abbreviations

IGFI: Indocyanine green fluorescence imaging
ICG: Indocyanine green
CRC: Colorectal cancer
NIR: Near-infrared
BMI: Body mass index
ASA: American Society of Anesthesiologists
CME: Complete mesocolic excision
CT: Computed tomography
